# Dialogue Based Early Detection—Development of a Novel Approach for Detection of Mental Health Problems Among Children in Daycare Centers

**DOI:** 10.3389/fpsyt.2022.696531

**Published:** 2022-02-18

**Authors:** Ingvar Bjelland, Maj-Britt Posserud, Gro Janne Wergeland

**Affiliations:** ^1^Division of Psychiatry, Department of Child and Adolescent Mental Health Services, Haukeland University Hospital, Bergen, Norway; ^2^Department of Clinical Medicine, University of Bergen, Bergen, Norway

**Keywords:** child, mental health, health promotion, screening, early detection, daycare center, kindergarten, parents

## Abstract

**Background:**

Among 1–7 years old children the worldwide prevalence of mental disorders is ~20%. Without treatment, the prognosis of such disorders in children is poor. Early intervention is estimated to result in a positive return. However, traditional screening to detect children at need is particularly challenging due to the concerns by false positives. The aim of the current study was to develop a more acceptable though effective method using the existing annual evaluation meetings between parents and teachers in a more systematic and goal directed way. The method should build on the teacher's and parents' complementary knowledge and perception of the child, and fit into the everyday routines in daycare centers.

**Method:**

During a period of 6 years, a developmental process aiming for a novel screening method was carried out in cooperation with eight Norwegian daycare centers. After conception of the idea, the framework of the Dialogue Based Early Detection including the first version of the Early Worry Questionnaire (EWQ) was constructed. An iterative process involving parents and teachers completing workshops and subsequent testing facilitated a re-modeling of the method.

**Results:**

In the resulting Dialogue Based Early Detection a 36-item version of EWQ was completed by both parents and teachers ahead of the annual parent-teacher meeting. During that meeting the participants should try to reach a consensus whether there was a concern, some uncertainty, or no worry for the child, and which appropriate actions should be taken for a possible follow up. Both parents and teachers reported that the EWQ supported them in verbalizing already existing worries for the child. Teachers reported that parents were better prepared and participated more actively in the evaluation meetings. However, some parents complained that there was too much focus on possible worries. During the testing, challenges of language development, conduct, emotional reactions, toileting, attention, and eating were detected among the children.

**Conclusion:**

The Dialogue Based Early Detection method was endorsed by both teachers and parents and holds promise as a tool for improving early awareness and identification of developmental and mental health problems of preschool children in daycare centers.

## Introduction

Mental disorders among children and adolescents represent a major public health challenge, with a worldwide prevalence of 13.4% (1). Among the youngest children (1–7 years old) the prevalence is even higher (20.1%) (2). The impact of common mental disorders among children such as ADHD as to major costs for both the individual and the society are wellknown, negatively affecting sick leave, increased mental and physical morbidity, alcohol and substance abuse, crime, relational problems, injuries, and suicide (3–9). Likewise, for children with anxiety disorders the impact is detrimental as well, with frequent experience of social, peer, and school related problems (10, 11).

It has been suggested that childhood mental health is the strongest predictor of adult wellbeing (12). Early detection and intervention are important to prevent secondary consequences of mental health problems, such as loss of education, ill health, and loss of parental earnings. Furthermore, early efforts are assumed to reduce socio-economic costs (13).

However, only a minority of children suffering from a mental disorder is detected and offered appropriate interventions, and younger children less often than older ones (14–17).

**Figure 1:**
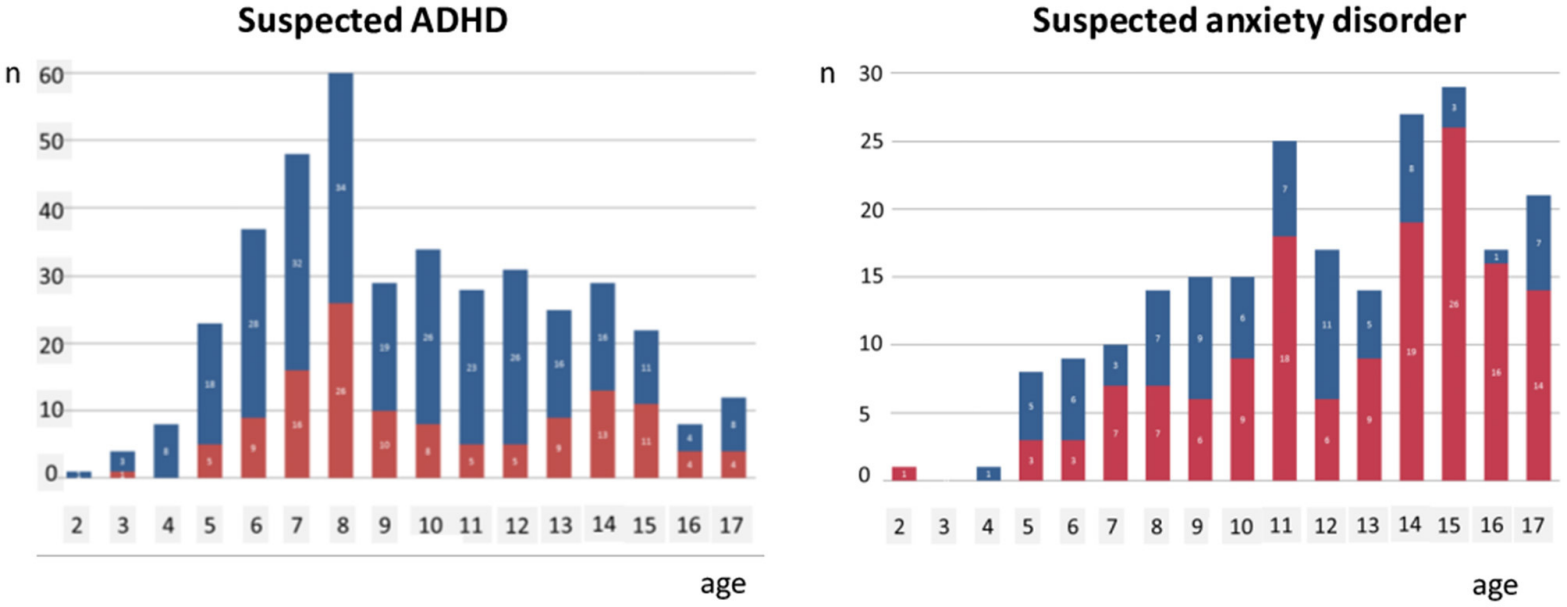
Age distribution of children referred to Child and Adolescent Mental Health Service in the catchment area of Haukeland University Hospital suspected for ADHD and anxiety disorders, respectively, during 2015 (blue bars: boys; red bars: girls).

For the individual child there may be years delay from the appearance of symptoms and difficulties to referral and intervention for these challenges. This aligns with clinical experience from Child and Adolescent Mental Health Service (CAMHS) where recurrently parents report that they were concerned for their child as early as 3–4 years of age. In 2015, the average age of referral to CAMHS in the catchment area of Haukeland University Hospital was far above school age for both suspected ADHD (9.7 years), for anxiety disorders (12.0 years), and higher for girls than for boys (Figure 1). This delay of several years most probably affects children's development where the lost years cannot be taken back. The CAMHS in Norway have reduced waiting time from referral to first consultation to 4 weeks for the youngest children. Rapid access to specialist services is, however, of limited use when the referral delay is several years.

Ideally, all children with significant emotional or behavioral difficulties should be detected and offered adequate developmental support to promote healthy emotional, social, and intellectual development and achieve optimal educational and social gains. For minor difficulties, preschool teachers or child health clinics may offer sufficient adaption and advice. For moderate difficulties, various services at the municipality level, such as special educationists, community psychologists, general practitioners, or physiotherapists, may offer a brief assessment and more specialized advice to preschool educators and parents. However, if the offered help at these levels fails or the difficulties are of a major kind, such as a suspected mental disorder, referral to CAMHS should be made.

According to our clinical experience, parents often report that their early concerns for their child were not taken seriously enough by the daycare center staff or the community nurse, who advised a “wait and see” approach. In Norway a daycare center (other translations: kindergarten, childcare center, nursery, nursery school, or preschool), is an all-day educational institution. The educational context is not school-like with classrooms and desks, but an environment facilitating ordinary play and other daily activities, nourishing normal physical, cognitive, emotional, and social development.

During their training, Norwegian preschool teachers learn much about normal development in children and how to promote it in their daily interaction with children. Together with the parents, as the closest caregivers, they have a fundamental impact on building resilience fostering good mental health in the individual child. In the daycare centers, children will meet different challenges in the social interactions with both other children and the staff. The teacher can guide the child to find a good balance between a positive self-representation and social respect to others. However, in general, preschool teachers show insecurity as to understanding and management of mental health challenges of the children when they appear. In Norway, as in most other countries, the health and educational sectors are separated, all from the governmental down to the elementary level. In their curriculum, preschool teacher students have minimal teaching about mental health, as this is considered a health matter. Further, in Norwegian municipalities systematic interaction and collaboration between these two sectors is uncommon.

While screening of mental health problems could be a way to improve access to help for young children, it is particularly challenging to screen for such problems in this age group. What is considered normality, covers a wide range of behavioral and emotional expressions. Moreover, symptoms of mental disorders are less specific than among older children. Many parents and preschool teachers dislike the objectifying approach of symptom assessment, as opposed to seeing, understanding and appreciating the child as a unique individual. Furthermore, traditional screening programs for behavioral or emotional problems in general populations of children are problematic, as such screening carries the downside of producing large numbers of false positive cases. They have thus been rightfully criticized by the users, i.e., preschool teachers and the parents of the children; claiming it pathologizes natural variation in behavior and in emotional reactions and generates unnecessary worry for healthy children.

Therefore, there is a clear need for better and more user-acceptable methods to identify children with a concerning development, ideally well in advance of starting school (18). After discussions with professional and academic communities of both preschool education and mental health, and search in the scientific literature, we could not find any pre-existing alternative to traditional screening aimed for early detection of mental health challenges in preschoolers. In Norway children start school the year they turn six, and ahead of that most children (>95%) from the age of 1 year attend a daycare center. Consequently, such an arena should be well-suited for a population-based screening of preschool children.

Hence, the aim of the current study was to develop a method to detect and manage possible worries for the individual child using the existing annual evaluation meetings between parents and teachers in daycare centers in a systematic and goal directed way. The method should build on the teacher's and parents' complementary knowledge and perception of the child and fit into the everyday routines. Such a method might have the potential to solve three major obstacles to develop efficient screening for child mental health problems—(i) the ethical problem of causing worry in healthy children, (ii) the time-consuming process of detailed symptomatic assessment, and (iii) being both sensitive and specific enough to fulfill the requirements as screening tool.

## Method

### Developmental Stages/Phases of DBED

Considering the challenges with the traditional screening of mental health problems, the first author was inspired by two different approaches to screening and clinical communication, respectively. The first, The Early Symptomatic Syndromes Eliciting Neurodevelopmental Clinical Examinations Questionnaire (ESSENCE-Q) (19) developed by Christopher Gillberg, is a screening tool that focuses on the parents' existing concerns for their child as opposed to assessing symptoms or functions. However, the ESSENCE–Q focuses specifically on neurodevelopmental disturbances and not on domains of emotional disturbances (19). To be used as a general screen, we aimed for a broader questionnaire and outlined The Early Worry Questionnaire (EWQ). Some of the ESSENCE-Q items were included, but we also added items from more traditional screening tools, such as the Achenbach's System of Empirically Based Assessment (ASEBA) (20), the Strengths and Difficulties Questionnaire (SDQ) (21), and the Attention, Behavior, Language, and Emotions **(**ABLE) (22). The aim was to cover motor, language, and general developmental issues in addition to behavior, emotional reactions, and some psychosomatic symptoms. We adapted the content of the specific items in these screening tools, not the exact wording, to make our wording appropriate for communication between parents and teachers. The second, was the Shared Decision Making approach (23). This approach focuses on user involvement, acknowledging the patient's unique competence of his/her own illness and life. It empowers the patient, giving the patient ownership to clinical decisions (“No decisions about me, without me”) and treatment. The Shared Decision Making approach was inspiring as it highlights the importance of the dialogue in dealing with challenging issues and decision-making. By combining these two approaches the idea of a novel screening method was conceived.

In Norwegian daycare centers, the parents have individual annual (or biannual) parent-teacher meeting to evaluate the child's wellbeing and development. We considered this meeting the optimal arena for screening, and outlined a two-step procedure for the purpose: To help the appropriate focus on the child's mental health, both parents and teachers should complete the EWQ as a preparation before the meeting. Parents should then participate actively in the meeting by giving their view of possible concerns for their child aiming for a common understanding of the child in the dialogue with the teacher. The method was named Dialogue Based Early Detection (DBED). To our knowledge, the use of such an approach has not been employed for screening purposes.

The further development was based on user involvement and consisted of an iterative process involving managers, teachers, and parents from eight daycare centers (Table 1).

**Table 1:** Development process of Dialogue Based Early Detection.

**Phase (year)**	**Elements**	**Outcome**
Origin (2015)	• ESSENCE-Q (19): Focus on parents' concern rather than assessment of symptoms • Shared decision making (23) • Daycare centers as arena of early detection of mental health challenges	• The idea of Dialogue Based Early Detection.
Start construction of EWQ (2016)	• Issues from ESSENCE-Q (19), ABLE (22), ASEBA (20), SDQ (21)	• First version of EWQ, 21 items.
Exposure to managers and teachers (2016)	• Separate meetings in four daycare centers for presentation and discussion	• Positive reception of the idea and outline. • Some suggestions of changes in EWQ.
Active user involvement in workshops (parents and teachers) to outline the first version of DBED (2017)	• Workshop 1: Assessment of every single item of EWQ • Workshop 2: How to present DBED for participants. How to use the results from the completed EWQs. How to conclude and follow up the outcome of the meetings • Workshop 3: Role play of parent-teacher meetings, sharing experiences of disagreement in such situations. Review of possible relevant supportive efforts. How to evaluate user satisfaction	• A modified version of EWQ, 33 items. • A guideline for implementation of DBED. • A list of relevant interventions for the various concerns. • User satisfaction questionnaires for parents and teachers, respectively.
Feasibility testing (2018–2019)	• Training of teachers of eight daycare centers by theory, role play, and following discussions. • Four centers participated in the testing during three rounds • Additionally four centers participated in the last round. • Approximately 300 parent-teacher meetings were recorded during the period • User satisfaction questionnaires from both parents and teachers. • Subsequent interviews of teachers	• Iterative testing resulted in numerous suggestions for improvements. • A further modified version of EWQ, 34 items. • From the final testing • De-emphasized focus on worry • Overall good user satisfaction (Tables 2, 3) • In 40% of children one or more areas of worry were detected, most often language problems.

First, we presented and discussed the proposed model for teachers and managers of four different daycare centers in separate meetings.

Second, we arranged three workshops with teachers and parents of children from the same four daycare centers for a more thorough review of the procedures in DBED. In the first workshop, every item of the EWQ was critically assessed as to both content and wording. In the second workshop, we discussed how to present the method and invite parents to participate in the coming testing. Further, we reflected on how to use the results from the completed EWQs in the parent-teacher meetings, included different challenges that might arise during the dialogue. Not least important, we shared ideas on how to conclude and follow up the outcome of the meeting. In the third workshop, the participants role played parent-teacher meetings and shared experiences of disagreement with parents as to reason for worry or not. Next, we went through possible supportive efforts relevant for the different kind of concerns, such as adaption and advice for minor concerns by preschool teachers or child health clinics, referral to special educationists, community psychologists, general practitioners, or physiotherapists for moderate concerns, or referral to child and adolescent mental health service where there is a major concern as to suspected mental disorder. In addition, we received feedback on how to evaluate user satisfaction of the method.

Third, we trained the teachers of eight daycare centers by theory, role play, and following discussions in advance of testing the method. The method was then tested in these centers repeatedly during 2018 and 2019 for children aged 3–6 years. Four centers participated in all three testing rounds, and four additional centers that had not participated in the preceding workshops, participated in the third and final round. During all rounds altogether 24 teachers participated and ~300 parent-teacher meetings were accomplished. After each round both parents and teachers were asked to complete a user satisfaction questionnaire, that ended with an open field for free text comments. The teachers were also interviewed by the first author (IB) in separate groups, one group for each center. They were asked by open questions to share their experiences with the method. The interviewer followed up by elaborative questions. Notes from the interviews were summarized by the first author. Every daycare center contributed with valuable suggestions for improvements of both wordings of the EWQ and details of the other procedures.

The four additional day care centers in the final round accomplished 153 parent-teacher meetings, from which data on user satisfaction and conclusions from the parent-teacher meetings were collected. The data protection official of Bergen municipality was consulted before this final testing, who concluded that a full ethical review at that stage of the project was not necessary. However, as a premise, all participating parents signed an informed consent and all information about the children (conclusions from the parent-teacher meetings) was delivered to us as aggregated data. Information about the individual child was stored safely in their respective day care center. All written feedback from teachers was voluntary and anonymous.

## Results

### General Feedback From Users

After the first presentation of the model teachers and managers of the four daycare centers responded overall positively. In their oral feedback they expressed a clear need for a more systematic approach and naming of specific mental health challenges among the children. In addition, they suggested more appropriate wordings or specifications of single items. For example, “language development” was differentiated to “pronunciation,” “vocabulary,” “use of language,” and “ability to understand what is said.” During the subsequent workshops suggestions for new items, such as “lack of eye contact,” came up spontaneously. In sum, 13 new items were added.

### The Resulting Versions of Dialogue Based Early Detection Method (DBED) and Early Worry Questionnaire (EWQ)

**Figure 2:**
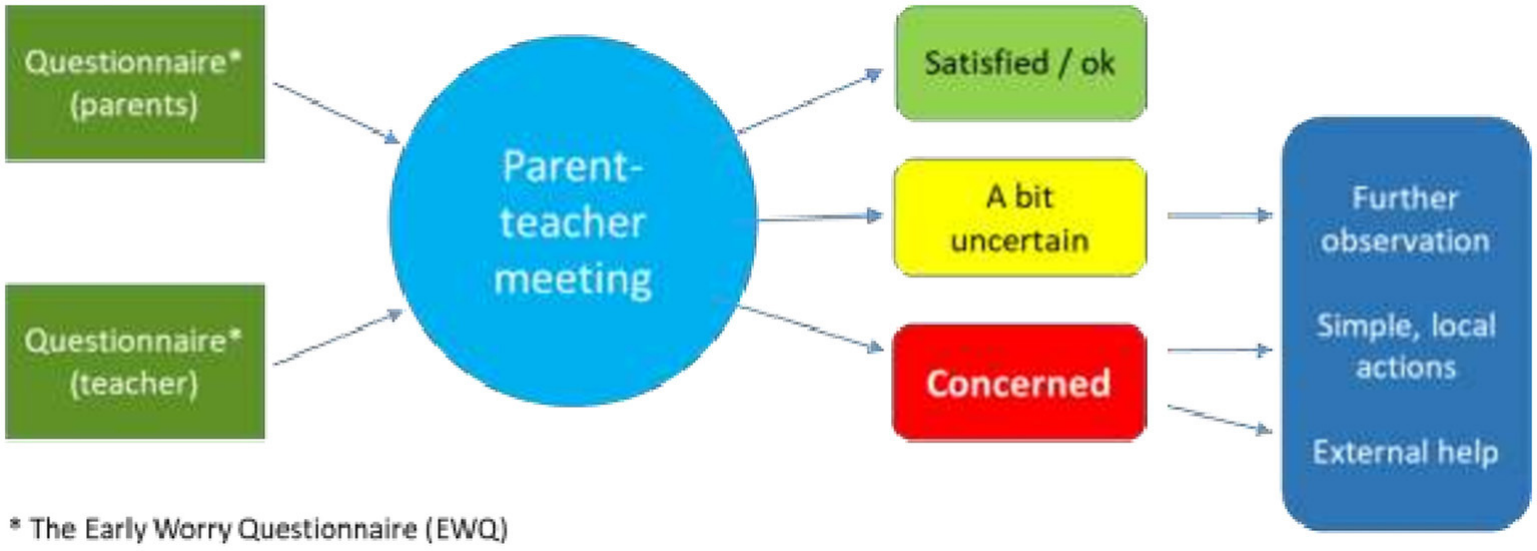
Dialogue based early detection.

The main element of DBED was the prepared and focused dialogue in the regular (annual or biannual) parent-teacher meeting. Both the parents and the teacher were expected to prepare for the dialogue by completing the EWQ prior to the meeting. The participants should try to reach a consensus whether there is a concern, some uncertainty, or no worry for the child, and which appropriate actions should be taken for a possible follow up (Figure 2). The DBED differs from traditional screening in four main ways; (1) by focusing on concerns for the child rather than symptoms, (2) consisting of a two-step process (i.e., questionnaire before a meeting), (3) including a dialogue between informants, and (4) not including a pre-set symptom cut-off. Instead, the outcome of the DBED is a consensus between parents and teachers after completing the DBED.

**Figure 3:**
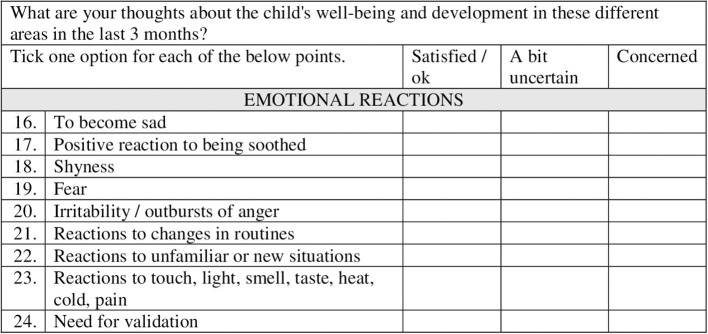
Example of some items in the Early Worry Questionnaire.

EWQ was finally presented as “About the child's wellbeing and development” (see Supplementary Material) and a section of this questionnaire is shown in Figure 3. It ended up with 36 items, where (1) two general questions focus on the child's development and wellbeing, (2) six items on language and mobility, (3) nine items on behavior, (4) nine items on emotional reactions, (5) five items on physical functions, and (6) five miscellaneous items not applicable in the former categories. The respondent is asked to give his/her judgement of the development and wellbeing during the last 3 months for each item, given three response options: (1) Satisfied/ok, (2) a bit uncertain, or (3) concerned. If the response is “a bit uncertain” or “concerned,” the respondent is requested to give a brief description of the challenge and his/her opinion why the child is struggling, given some specific categories related to conditions in the daycare center, at home, or others.

### Acceptability of the EWQ and the DBED

Completed user satisfaction questionnaires from the final testing round were received from 11 teachers (65% response rate) and parents of 110 children (72% response rate). The results showed that in general the method was well-accepted (Tables 2, 3). During the interviews the teachers generally reported that the EWQ supported them in verbalizing already existing worries for the child. They also consistently reported that parents were better prepared and participated more actively in the parent-teacher meetings than before. Most teachers experienced that some parents found it easier to express their incipient concern for their child. Coincidently, some teachers reported that they were engaged into more counseling addressing such concerns. This was a positive experience as their educational competence most often was sufficient to consider whether a specific behavior or developmental task was within the normal range of variation. However, comments in the free text fields in the questionnaires indicated that some parents and teachers disliked the focus on concern or worry for the child. This concern led to a revision of title and response options. The original title of the delivered questionnaires was “Are you concerned for the child's development and wellbeing?” and the response options for each item were “Yes,” “Uncertain,” “Somewhat,” or “No,” Changing the title to “About the child's wellbeing and development” and the response options to “Satisfied/Ok,” “A bit uncertain,” or “Concerned,” alleviated the objections significantly.

**Table 2:** User satisfaction, parents questionnaires completed after the parent-teacher meeting after final testing round in 2019 (*N* = 110).

		**Strongly agree**	**Somewhat agree**	**Somewhat disagree**	**Strongly disagree**	**Don't know**
1	It was easy to understand why I was/we were supposed to fill out the questionnaire	66	24	2	1	0
2	It was difficult to fill out the questionnaire	9	10	12	70	0
3	Speaking with the teacher during the review of the questionnaire in the parent-teacher conference was a good experience	97	10	1	1	1
4	I/we did not think the questionnaire was useful	9	4	16	81	0
5	I/we did not think the conversation was useful	9	2	7	92	0
6	Such a method should be used in connection with all parent-teacher conferences	63	33	3	2	10

**Table 3:** User satisfaction, teacher questionnaires completed after final testing round in 2019 (*N* = 11).

		**Strongly agree**	**Somewhat agree**	**Somewhat disagree**	**Strongly disagree**	**Don't know**
1	It was difficult to fill out the questionnaire	0	1	3	7	0
2	It was easy to introduce the questionnaire in the meeting	5	4	2	0	0
3	The questionnaire was an obstacle for a good dialogue with the parents	0	0	2	9	0
4	This method was helpful to clarify together with the parents if there was a reason to be worried for the child or not	8	3	0	0	0
5	It was difficult to summarize the present worries for the child	0	0	4	7	0
6	This method made it easier to reach relevant actions for the child together with the parents	6	5	0	0	0
7	This method did not contribute to a good dialogue with the parents about possible worries for the child	0	0	2	9	0
8	Such a method should be used in connection with all parent-teacher conferences	4	6	0	0	0

### Pilot Screening Results of the DBED

In the final testing, conclusions were reported from all 153 parent-teacher meetings. In 61 (39.9%) of these cases the dialogue concluded with “A bit uncertain” or “Concerned” in at least one item. Most often “language” (*n* = 31, 20.3%) was the matter of concern, then “conduct” (*n* = 15, 9.8%), “emotional reactions” (*n* = 5, 3.3%), “peeing and pooping” (*n* = 4, 2.6%), “attention” (*n* = 3, 2.0%), and “eating” (*n* = 3, 2.0%). External services involved were special educationalists, speech therapist, physiotherapist, GP, child, and adolescent mental health service, pediatrics, and child welfare. As a child and adolescent psychiatrist the first author offered consultations for single case guidance, which, however, was not necessary.

## Discussion

The Dialogue Based Early Detection (DBED) is a novel method for early detection of mental health challenges among children in daycare centers. The method consists of (1) the Early Worry Questionnaire (EWQ) to be completed by parents and teachers before (2) the discussion at the regular parent-teacher conference. During testing of the method under everyday conditions, the DBED has been well-accepted by parents and teachers, and also seems to function well as a screen for mental health and developmental problems. Preparation before the parent-teacher meetings by the EWQ seemed to facilitate a more active involvement of the parents in the dialogue, which next challenged the teachers to play a more counseling role in the meeting.

The DBED fills a void in providing parents and preschool teachers with a tool to focus on behavior and symptoms that may be early signs of mental health challenges. To this aim, the DBED contains more novel elements: First, the utilization of the regular teacher-parent meetings to focus on the child's wellbeing and mental health. Second, the systematic and multi-domain Early Worry Questionnaire developed in cooperation with parents and teachers taking into account what items and formulations are suitable in the context of daycare centers. Third, active involvement of the parents in the assessment of their child by completing the same questionnaire (EWQ) as the teacher, thereby empowering their role in the dialogue concerning the development and wellbeing of their child. With the two-step process, focusing on concerns and the consensus decision reached through the dialogue, the DBED holds promise as a sensitive yet specific screening tool. This is essential for the next step, i.e., offering the right help to the right children at the right time. Our experience from the present developmental project is that the daycare centers is a virginal field for early promotion of mental health. However, here is a need for more mental health competence among teachers and a more systematic approach for early detection and intervention. This development is hampered by the barriers between the health sector and the educational sector at all levels. Many teachers express a need for more knowledge of mental health problems and how to adapt the education for vulnerable children. The health sector on the other hand, may be too focused on pathology and deviances, as was clear from teachers' and parents' reactions to the initial wordings in the EWQ. The pathology approach is not adequate in a general population setting such as a daycare center, where most children are healthy.

The focus on pathology and problems was the main objection to the method from both parents and teachers. This lead to rephrasing of many of the items to better fit an educational setting. The inconvenience of extra time and effort spent using the DBED was viewed by the teachers as worth the investment.

Some teachers were concerned that the DBED approach might overlook causes of distress and poor development of children. However, in EWQ the respondents are invited to suggest possible causes for the reported concern, given examples such as serious events or situations at home or otherwise in the family (illness, accidents, death, separation, finances, living arrangements) and difficulties in relation to the daycare center (bullying by other children, difficulties in relation to particular adults). Nevertheless, very few respondents made such suggestions. A possible improvement of the EWQ might be to introduce a few check boxes with some broad “cause categories” supplemented with space for free text.

In ~40% of the parent-teacher meetings the conclusion was that there was one or more areas of worry for the child, with language problems being the most frequent worry. This indicates the method is sensitive to identify developmental concerns. The most salient question is, however, whether the implementation of the DBED really detects the right children, providing earlier access to support and intervention for them, and, ultimately, have a positive effect on the individual child's future mental health. Future studies will examine the DBED in terms of (1) its feasibility more thoroughly in everyday life in ordinary daycare centers, (2) its case-finding properties, (3) how it influences early interventions in the ordinary public services, and (4) whether it will have a positive influence on the children's future mental health.

## Conclusion

The novel Dialogue Based Early Detection (DBED) method was endorsed by both teachers and parents and holds promise as a tool for improving early identification of mental health problems of preschool children in daycare centers. It differs from traditional screening methods due to its two-stage procedure, its focus on the concern for the child rather than symptoms, and finally in the questionnaire prepared dialogue between teacher and parents before a consensus conclusion is reached. However, further evaluation of the method is imperative.

## Data Availability Statement

The original contributions presented in the study are included in the article/Supplementary Material, further inquiries can be directed to the corresponding author/s.

## Ethics Statement

Ethical review and approval was not required for the study on human participants in accordance with the local legislation and institutional requirements. Written informed consent to participate in this study was provided by the participants' legal guardian/next of kin.

## Author Contributions

IB created the idea of the Dialogue Based Early Detection and initiated and led the development process described in the present paper and wrote the first outline of the draft, and led the following writing process. GW and M-BP have contributed with intellectual input during the development of the present method and participated in the writing process of the paper. All authors contributed to the article and approved the submitted version.

## Conflict of Interest

GW reports receiving consulting fees and lecture fees from Medice. M-BP reports receiving no fees as a member of the scientific advisory board of Takeda/Neurim for Slenyto. The remaining author declares that the research was conducted in the absence of any commercial or financial relationships that could be construed as a potential conflict of interest.

## Publisher's Note

All claims expressed in this article are solely those of the authors and do not necessarily represent those of their affiliated organizations, or those of the publisher, the editors and the reviewers. Any product that may be evaluated in this article, or claim that may be made by its manufacturer, is not guaranteed or endorsed by the publisher.
