# Supplementary material for: Dialogue Based Early Detection—Development of a Novel Approach for Detection of Mental Health Problems Among Children in Daycare Centers
Source: Front Psychiatry. 2022 Feb 18;13:696531. doi: 10.3389/fpsyt.2022.696531 (PMC8896884; doi:10.3389/fpsyt.2022.696531)
Supplement: Supplementary file 1 [file Data_Sheet_1.docx]

The Early Worry Questionnaire (EWQ):

**ABOUT THE CHILD'S WELL-BEING AND DEVELOPMENT**

| Child's name |  |  |  |
| --- | --- | --- | --- |

| Completed (date) |  | Boy |  |  | Girl |  | Age  (years) |  |  |
| --- | --- | --- | --- | --- | --- | --- | --- | --- | --- |

| Who completed the questionnaire? (tick one of the options) | |
| --- | --- |
|  | Parents/guardians (the person(s) the child resides with most of the time) |
|  | Pre-school teacher |

Please complete this questionnaire – in preparation for the upcoming parent-teacher meeting

**A child can of course not be fully described – or understood – by a completed questionnaire!**

This questionnaire is only meant as a tool to facilitate the sharing of thoughts about specific issues important for the child’s well-being and development.

| **Overall** | Satisfied / ok | A bit uncertain | Concerned |
| --- | --- | --- | --- |
| What are your thoughts on the child's development in the last 3 months? |  |  |  |
| What are your thoughts on the child's well-being in the last 3 months? |  |  |  |

**Regarding these different areas**, what are your thoughts on the child's well-being and development in the last 3 months?

| Tick one option for each of the points below. | | Satisfied / ok | A bit uncertain | Concerned |
| --- | --- | --- | --- | --- |
| LANGUAGE AND MOTOR SKILLS | | | | |
| 1. | Pronunciation of words |  |  |  |
| 2. | Vocabulary |  |  |  |
| 3. | Use of language / sentence construction |  |  |  |
| 4. | Ability to understand what is said |  |  |  |
| 5. | Gross motor skills: How the child uses his/her body. |  |  |  |
| 6. | Fine motor skills: E.g. cutting, drawing, threading beads, using a knife / fork |  |  |  |
| BEHAVIOUR | | | | |
| 7. | Taking the initiative to play with other children |  |  |  |
| 8. | Being considerate towards other children |  |  |  |
| 9. | Variation in type of activity / play |  |  |  |
| 10. | Listening |  |  |  |
| 11. | Level of activity (appropriate or too low/high) |  |  |  |
| 12. | Engaging in an activity over time |  |  |  |
| 13. | Not becoming distracted |  |  |  |
| 14. | Eye contact |  |  |  |
| 15. | Mimicry (facial expressions displaying reactions/feelings/emotions |  |  |  |
| EMOTIONAL REACTIONS | | | | |
| 16. | Becoming sad |  |  |  |
| 17. | Positive reaction to being soothed |  |  |  |
| 18. | Shyness |  |  |  |
| 19. | Fear |  |  |  |
| 20. | Irritability / outbursts of anger |  |  |  |
| 21. | Reactions to changes in routines |  |  |  |
| 22. | Reactions to unfamiliar or new situations |  |  |  |
| 23. | Reactions to touch, light, smell, taste, heat, cold, pain |  |  |  |
| 24. | Need for affirmation |  |  |  |
| BODILY FUNCTIONS | | | | |
| 25. | Vision |  |  |  |
| 26. | Hearing |  |  |  |
| 27. | Sleep (only to be answered by parents/guardians) |  |  |  |
| 28. | Eating habits |  |  |  |
| 29. | Peeing and pooping |  |  |  |
| OTHER | | | | |
| 30. | Other issues |  |  |  |

If **A bit uncertain** or **Concerned** was the answer to one or more of the above questions, please write down a few lines about this here:

……………………………………………………………………………………………………………

……………………………………………………………………………………………………………

……………………………………………………………………………………………………………

If you have any thoughts about **why** the child is having difficulties in one or more areas, please write a few lines about this here. Examples: Serious events or situations at home or otherwise in the family (illness, accidents, death, separation, finances, living arrangements, etc...). Difficulties in relation to nursery/day care center (bullying by other children, difficulties in relation to particular adults, etc...). Other things?

……………………………………………………………………………………………………………

……………………………………………………………………………………………………………

……………………………………………………………………………………………………………

| Have you witnessed any of the following in the child in the last 3 months? | | No | A few times | Often |
| --- | --- | --- | --- | --- |
| 30. | Hitting, biting, shoving, threatening (more than usual for the age of the child) |  |  |  |
| 31. | Uncontrolled movements |  |  |  |
| 32. | Loss of skills / more immature functioning |  |  |  |
| 33. | “Fits” where the child appears distant or strange |  |  |  |
| 34. | Stomach ache or head ache |  |  |  |

If **Often** or **A few times** was the answer to one or more of the above questions, please write a few lines about this here:

……………………………………………………………………………………………………………

……………………………………………………………………………………………………………

……………………………………………………………………………………………………………
